# A million-cow genome-wide association study of productive life in U.S. Holstein cows

**DOI:** 10.1186/s12711-024-00935-1

**Published:** 2024-09-26

**Authors:** Zuoxiang Liang, Dzianis Prakapenka, Hafedh B. Zaabza, Paul M. VanRaden, Curtis P. Van Tassell, Yang Da

**Affiliations:** 1 Department of Animal Science, University of Minnesota, Saint Paul, MN 55108 USA; 2 Animal Genomics and Improvement Laboratory, USDA-ARS, Beltsville, MD 20705 USA

## Abstract

**Background:**

Productive life (PL) of a cow is the time the cow remains in the milking herd from first calving to exit from the herd due to culling or death and is an important economic trait in U.S. Holstein cattle. The large samples of Holstein genomic evaluation data that have become available recently provided unprecedented statistical power to identify genetic factors affecting PL in Holstein cows using the approach of genome-wide association study (GWAS).

**Methods:**

The GWAS analysis used 1,103,641 Holstein cows with phenotypic observations on PL and genotypes of 75,282 single nucleotide polymorphism (SNP) markers. The statistical tests and estimation of SNP additive and dominance effects used the approximate generalized least squares method implemented by the EPISNPmpi computer program.

**Results:**

The GWAS detected 5390 significant additive effects of PL distributed over all 29 autosomes and the X–Y nonrecombining region of the X chromosome (Chr31). Two chromosome regions had the most significant and largest cluster of additive effects, the *SLC4A4-GC*-*NPFFR2* (SGN) region of Chr06 with pleiotropic effects for PL, fertility, somatic cell score and milk yield; and the 32–52 Mb region of Chr10 with peak effects for PL in or near *RASGRP1* with many important immunity functions. The dominance tests detected 38 significant dominance effects including 12 dominance effects with sharply negative homozygous recessive genotypes on Chr18, Chr05, Chr23 and Chr24.

**Conclusions:**

The GWAS results showed that highly significant genetic effects for PL were in chromosome regions known to have highly significant effects for fertility and health and a chromosome region with multiple genes with reproductive and immunity functions. SNPs with rare but sharply negative homozygous recessive genotypes for PL existed and should be used for eliminating heifers carrying those homozygous recessive genotypes.

**Supplementary Information:**

The online version contains supplementary material available at 10.1186/s12711-024-00935-1.

## Background

Productive life (PL) of a cow is the time the cow remains in the milking herd from first calving to exit from the herd due to culling or death expressed as the difference in months of productivity compared to the breed base, and is a longevity trait [1]. PL is associated with profitability of dairy farms and the main contributing factors to PL were health, ability to conceive, and performance in milk production [2]. The current selection index (net merit) in U.S. Holstein cattle assigns the third largest relative emphasis to PL after fat and protein yields among the sixteen traits in the index [3, 4], indicating an important role of PL in Holstein cattle breeding. Genome-wide association studies (GWAS) is a powerful approach to investigate the association between genetic factors and the phenotypes, and several GWAS for PL in Holstein cattle have been reported [5–8]. However, those reports were based on different Holstein populations with different sample sizes up to fewer than 80,000 cows and generally lacked mutual confirmation of significant genetic effects. Therefore, additional studies, particularly those with large samples, are needed for building consensus about genetic factors affecting PL. The sample size of U.S. Holstein cows for genomic evaluation has been increasing rapidly [9], surpassing one million by the end of 2022, and this data provide an excellent opportunity for GWAS for PL. Such large sample sizes should provide much greater statistical power than available from previous GWAS reports for detecting genetic variants affecting PL, and recent studies using such large samples produced high confidence evidence for genetic factors affecting four reproduction traits and fat percentage [10–12]. The purpose of this study was to obtain high-confidence evidence for genetic factors affecting PL from GWAS using the U.S. Holstein million-cow genomic evaluation resources.

## Methods

### Holstein population and SNP data

The Holstein population in this study had 1,103,641 cows with phenotypic observations on PL and genotypes of 78,964 original and imputed SNPs. The phenotypic values used in the GWAS analysis were the phenotypic residuals after removing fixed non-genetic effects available from the December 2023 U.S. Holstein genomic evaluation by Council on Dairy Cattle Breeding (CDCB). The SNP genotypes were from 32 SNP chips with various densities and were imputed to 78,964 SNPs using the FindHap algorithm [13] as a routine procedure for genomic evaluation by CDCB [14]. The SNP genotyping quality control by CDCB had checks and requirements at the individual and SNP levels, including call rate, parent-progeny conflicts, sex verification using X-specific SNPs, and Hardy–Weinberg equilibrium [15, 16]. In addition, we applied minor allele frequency (MAF) of 5% for SNP filtering in this study. With the requirement of 5% MAF, the number of SNPs for the GWAS analysis was 75,282. The threshold p-value for declaring significant effects using the Bonferroni correction with 0.05 genome-wide false positives for 75,282 SNPs was 10^−8^, or log_10_(1/p) = 8. The SNP and gene positions were those from the ARS-UCD1.3 cattle genome assembly [17]. Genes containing or in the proximity of highly significant additive and dominance SNP effects were identified as candidate genes affecting PL.

### GWAS analysis

The GWAS analysis used an approximate generalized least squares (AGLS) method. The AGLS method combines the least squares (LS) tests implemented by EPISNP1mpi [18, 19] with the estimated breeding values from routine genetic evaluation using the entire U.S. Holstein population. The statistical model was:

1 $$\mathbf{y}={\varvec{\upmu}}1+{\mathbf{X}}_{\mathbf{g}}\mathbf{g}+\mathbf{Z}\mathbf{a}+\mathbf{e}=\mathbf{X}\mathbf{b}+\mathbf{Z}\mathbf{a}+\mathbf{e},$$

where **y** = column vector of phenotypic deviation after removing fixed nongenetic effects such as heard-year-season (termed as ‘yield deviation’ for any trait) using a standard procedure for the CDCB/USDA genetic and genomic evaluation; µ = common mean; **1 = **column vector of 1’s; **g** = column vector of genotypic values of the three SNP genotypes; **X**_g_ = model matrix of **g** with ‘1’ and ‘0’ indicator values; and $\mathbf{b}=(\upmu ,\mathbf{g}\mathbf{^{\prime}})\mathbf{^{\prime}}$, **X** = (**1**, **X**_g_); **a** = column vector of additive polygenic values; **Z** = model matrix of **a**; and **e** = column vector of random residuals. The first and second moments of Eq. (1) are: E(**y**) = **Xb** and $\text{var}\left(\mathbf{y}\right)=\mathbf{V}=\mathbf{Z}\mathbf{G}{\mathbf{Z}}^{\mathbf{^{\prime}}}+\mathbf{R}={\upsigma }_{\text{a}}^{2}\mathbf{Z}\mathbf{A}{\mathbf{Z}}^{\mathbf{^{\prime}}}+{\upsigma }_{\text{e}}^{2}\mathbf{I}$, where ${\upsigma }_{\text{a}}^{2}$ = additive variance, **A** = additive relationship matrix, and ${\upsigma }_{\text{e}}^{2}$= residual variance. The problem of estimating the **b** vector that includes SNP genotypic values in Eq. (1) is that it requires inverting **V** if the generalized least squares (GLS) method is used or solving the mixed model equations (MME) [20], as shown by Eqs. 2 and 3 below. Either the GLS or MME method for each of the genome-wide SNPs is computationally challenging for our sample size. To avoid these computing difficulties, the GWAS used the method of approximate GLS (AGLS) that replaces the polygenic additive values (**a**) with the best linear unbiased prediction based on pedigree relationships [21]. The AGLS method is based on the following results:

2 $$\hat{\mathbf{b}}={(\mathbf{X}^\prime{\mathbf{V}}^{-1}\mathbf{X})}^{-}{\mathbf{X}}^{\prime}{\mathbf{V}^{-1}}\mathbf{y}$$

3 $$\begin{aligned}\hat{\mathbf{b}}={\left({\mathbf{X}}^{\prime}{\mathbf{R}}^{-1}\mathbf{X}\right)}^{-}\left({\mathbf{X}}^{{\prime}}{\mathbf{R}}^{-1}\mathbf{y}-{\mathbf{X}}^{\prime}{\mathbf{R}}^{-1}\mathbf{Z}\hat{\mathbf{a}}\right)\\={\left({\mathbf{X}}^{\prime}\mathbf{X}\right)}^{-}\mathbf{X}^{\prime}\left(\mathbf{y}-\mathbf{Z}\hat{\mathbf{a}}\right)={\left({\mathbf{X}}^{\prime}\mathbf{X}\right)}^{-}\mathbf{X}^{\prime}{\mathbf{y}}^{\boldsymbol{*}}\end{aligned}$$

where ${\mathbf{y}}^{\boldsymbol{*}}=\mathbf{y}-\mathbf{Z}\hat{\mathbf{a}}$ and $\hat{\mathbf{a}}$ is the best linear unbiased prediction (BLUP) of **a**. Equation (2) is the GLS solution, and Eq. (3) is the MME solution of **b**. These two equations yield identical results, and $\hat{\mathbf{b}}$ from either equation is termed the best linear unbiased estimator (BLUE) [20]. If $\hat{\mathbf{a}}$ is known, the LS version of BLUE given by Eq. (3) is computationally efficient relative to the GLS of Eq. (2) that requires the **V** inverse or the joint MME solutions of $\hat{\mathbf{b}}$ and $\hat{\mathbf{a}}$. The AGLS method uses two approximations. The first approximation is to use $\widetilde{\mathbf{a}}$ from routine genetic evaluation as an approximation of $\hat{\mathbf{a}}$ in Eq. (3):

4 $$\hat{\mathbf{b}}={\left({\mathbf{X}}^{\prime}\mathbf{X}\right)}^{-}\mathbf{X}^{\prime}\left(\mathbf{y}-\mathbf{Z}\widetilde{\mathbf{a}}\right)={\left({\mathbf{X}}^{\prime}\mathbf{X}\right)}^{-}\mathbf{X}^{\prime}{\mathbf{y}}^{\boldsymbol{*}}$$

where ${\mathbf{y}}^{\boldsymbol{*}}=\mathbf{y}-\mathbf{Z}\widetilde{\mathbf{a}}$, and $\widetilde{\mathbf{a}}$ is the column vector of 2(PTA) with PTA being the predicted transmitting ability from the routine genetic evaluation. Equation (4) achieves the benefit of sample stratification correction from mixed models using pedigree relationships without the computing difficulty of inverting **V** or the joint MME solutions of $\hat{\mathbf{b}}$ and $\hat{\mathbf{a}}$ for every SNP. The second approximation of the AGLS approach is the t-test using the LS rather than the GLS formula of the t-statistic to avoid using the **V** inverse in the GLS formula. The significance tests for additive and dominance SNP effects used the t-tests of the additive and dominance contrasts of the estimated SNP genotypic values [18, 22]. The t-statistic of the AGLS was calculated as:

5 $${\text{t}}_{\text{j}}=\frac{\left|{\text{L}}_{\text{j}}\right|}{\sqrt{\text{var}({\text{L}}_{\text{j}})}}=\frac{\left|{\mathbf{s}}_{\text{j}}\hat{\mathbf{g}}\right|}{\text{v}\sqrt{{\mathbf{s}}_{\text{j}}{(\mathbf{X}}^{\prime}{\mathbf{X})}_{\text{gg}}^{-}{\mathbf{s}}_{\text{j}}^{\prime}}},\text{ j}=\text{a},\text{d}$$

where L_j_ = additive or dominance contrast; $\sqrt{\text{var}({\text{L}}_{\text{j}})}$= standard deviation of the additive or dominance contrast; ${\mathbf{s}}_{\text{a}}$**=** row vector of additive contrast coefficients = $$\left[\begin{array}{ccc}{\text{P}}_{11}/{\text{p}}_{1}& 0.5{\text{P}}_{12}({\text{p}}_{2}-{\text{p}}_{1})/({\text{p}}_{1}{\text{p}}_{2})& {-\text{P}}_{22}/{\text{p}}_{2}\end{array}\right]$$; ${\mathbf{s}}_{\text{d}}$= row vector of dominance contrast coefficients = $$\left[\begin{array}{ccc}-0.5& 1& 0.5\end{array}\right]$$; ${\text{v}}^{2}=\left(\mathbf{y}-\mathbf{X}\hat{\mathbf{b}}\right)^\prime(\mathbf{y}-\mathbf{X}\hat{\mathbf{b}})/(\text{n}-\text{k})$= estimated residual variance; $\hat{\mathbf{g}}$ = column vector of the AGLS estimates of the three SNP genotypic effects of ${\text{g}}_{11}$, ${\text{g}}_{12}$, and ${\text{g}}_{22}$ from Eq. (4); ${(\mathbf{X}^{\prime}\mathbf{X})}_{\text{gg}}^{-}$ = submatrix of ${\left({\mathbf{X}}^{\prime}\mathbf{X}\right)}^{-}$ corresponding to $\hat{\mathbf{g}}$; and where ${\text{p}}_{1}$ = frequency of ${A}_{1}$ allele, ${\text{p}}_{2}$= frequency of ${A}_{2}$ allele of the SNP, ${\text{P}}_{11}$ = frequency of ${A}_{1}{A}_{1}$ genotype, ${\text{P}}_{12}$ = frequency of ${A}_{1}{A}_{2}$ genotype, ${\text{P}}_{22}$ = frequency of ${A}_{2}{A}_{2}$ genotype, n = number of observations, and k = rank of **X**. The formula of ${\mathbf{s}}_{\text{a}}$ defined above allows Hardy–Weinberg disequilibrium [22], and simplifies to $$\left[\begin{array}{ccc}{\text{p}}_{1}& {\text{p}}_{2}-{\text{p}}_{1}& -{\text{p}}_{2}\end{array}\right]$$ under Hardy–Weinberg equilibrium.

Additive effects of each SNP were estimated using three measures, the average effect of gene substitution, allelic mean, and allelic effect of each allele based on quantitative genetics definitions [22, 23]. The allelic mean (${\upmu }_{\text{i}}$), the population mean of all genotypic values of the SNP (μ), the allelic effect (${\text{a}}_{\text{i}}$), and the average effect of gene substitution of the SNP (α) are:

6 $${\upmu }_{1}={\text{P}}_{11.1}{\text{g}}_{11}+0.5{\text{P}}_{12.1}{\text{g}}_{12}$$

7 $${\upmu }_{2}={0.5\text{P}}_{12.2}{\text{g}}_{12}+{\text{P}}_{22.2}{\text{g}}_{22}$$

8 $$\upmu =\sum\limits_{\text{i}=1}^{2}{\text{p}}_{\text{i}}{\upmu }_{\text{i}}$$

9 $${\text{a}}_{\text{i}}={\upmu }_{\text{i}}-\upmu ,\text{ i}=1, 2$$

10 $${\alpha }={\text{L}}_{\text{a}}={\mathbf{s}}_{\text{a}}\hat{\mathbf{g}}={\text{a}}_{1}-{\text{a}}_{2}={\upmu }_{1}-{\upmu }_{2}$$

where ${\text{P}}_{11.1}={\text{P}}_{11}/{\text{p}}_{1}$, ${\text{P}}_{12.1}={\text{P}}_{12}/{\text{p}}_{1}$, ${\text{P}}_{12.2}={\text{P}}_{12}/{\text{p}}_{2}$, and ${\text{P}}_{22.2}={\text{P}}_{22}/{\text{p}}_{2}$. The additive effect measured by the average effect of gene substitution of Eq. (10) is the distance between the two allelic means or effects of the same SNP and is the fundamental measure for detecting SNP additive effects as shown by the t-statistic of Eq. (5). The allelic effects defined by Eq. (9) provide an understanding of the effect size and direction of each allelic effect.

The reporting of additive effects combined statistical significance based on Eq. (5) and the allelic effects defined by Eq. (9). The statistical significance identified SNPs with significant additive effects and the allelic effects provided an understanding of the significant effects in terms of the size and direction of each allelic effect. This integrated reporting of statistical significance and allelic effects was also applied to reporting dominance effects where the reporting combined statistical significance and genotypic effects in terms of dominance deviations and the genotypic averages of the original phenotypic values.

The dominance effect of each SNP was estimated as the dominance contrast of $\hat{\mathbf{g}}$ from Eq. (4):

11 $$\updelta ={\text{L}}_{\text{d}}={\text{d}}_{12}-{(\text{d}}_{11}+{\text{d}}_{22})/2={\text{g}}_{12}-{(\text{g}}_{11}+{\text{g}}_{22})/2$$

where ${\text{g}}_{\text{ij}}$ is the AGLS estimates of SNP genotypic value from Eq. (4) (i, j = 1, 2), and ${\text{d}}_{\text{ij}}$ is the dominance value (dominance deviation) of the ${A}_{\text{i}}{A}_{\text{j}}$ SNP genotype:

12 $${\text{d}}_{\text{ij}}={\text{g}}_{\text{ij}}-\upmu - {\text{a}}_{\text{i}}-{\text{a}}_{\text{j}}$$

The degree of dominance of a dominance effect for a quantitative trait is defined in analogy to the example for fitness genotypes (Fig. 2.1 in Falconer and Mackay) [23]: the dominance effect is overdominance if the fitness of the heterozygous genotype is more extreme than either homozygous genotype, partial dominance if the heterozygous genotype is between the two homozygous genotypes, or complete dominance if the heterozygous genotype is the same as one of the homozygous genotypes. In this study, the degree of dominance is measured using dominance values (deviations) of Eq. (12). The dominance effect is overdominance if the dominance value of the heterozygous genotype is more extreme than that of either homozygous genotype: $|{\text{d}}_{12}|>{|\text{d}}_{11}|$ and ${|\text{d}}_{12}|>{|\text{d}}_{22}|$; partial dominance if the dominance value of the heterozygous genotype is between the dominance values of the two homozygous genotypes: ${\text{d}}_{11}$ < ${\text{d}}_{12}<{\text{d}}_{22}$ or ${\text{d}}_{11}$ > ${\text{d}}_{12}>{\text{d}}_{22}$; or complete dominance if the dominance value of the heterozygous genotype is the same as the dominance value of one of the two homozygous genotypes: ${\text{d}}_{11}$ ≠ ${\text{d}}_{12}={\text{d}}_{22}$ or ${\text{d}}_{11}$ = ${\text{d}}_{12}\ne {\text{d}}_{22}$. Due to random variations of the quantitative trait, the ‘=’ sign for complete dominance in general could not be exact. An allele is defined as a dominant allele if this allele neutralizes the effect of the alternative allele completely or partially when in heterozygous status. Similarly, an allele is defined as a recessive allele if the effect of this allele is neutralized by the alternative allele completely or partially when in heterozygous status.

To evaluate the impact of sharply negative homozygous recessive genotypes, a measure of negative impact was calculated as the difference between mean phenotypic values of cows with the homozygous recessive genotypes and the mean values of the other two genotypes, the homozygous dominant genotype, and the heterozygous genotype:

13 $$\text{NI}={\text{y}}_{\text{rr}}-({\text{y}}_{\text{rd}}+{\text{y}}_{\text{dd}})/2$$

where NI = negative impact of the homozygous recessive genotype. The genotypic average of the phenotypic values were denoted by ${\text{y}}_{\text{rr}}$, ${\text{y}}_{\text{rd}}$ and ${\text{y}}_{\text{dd}}$, where ${\text{y}}_{\text{rr}}$ = the average of the original phenotypic values of cows with the homozygous recessive genotype, ${\text{y}}_{\text{rd}}$ = the average of the original phenotypic values of cows with the heterozygous genotype, and ${\text{y}}_{\text{dd}}$ = the average of the original phenotypic values of cows with the homozygous dominant genotype of the SNP.

## Results and discussion

**Fig. 1:**
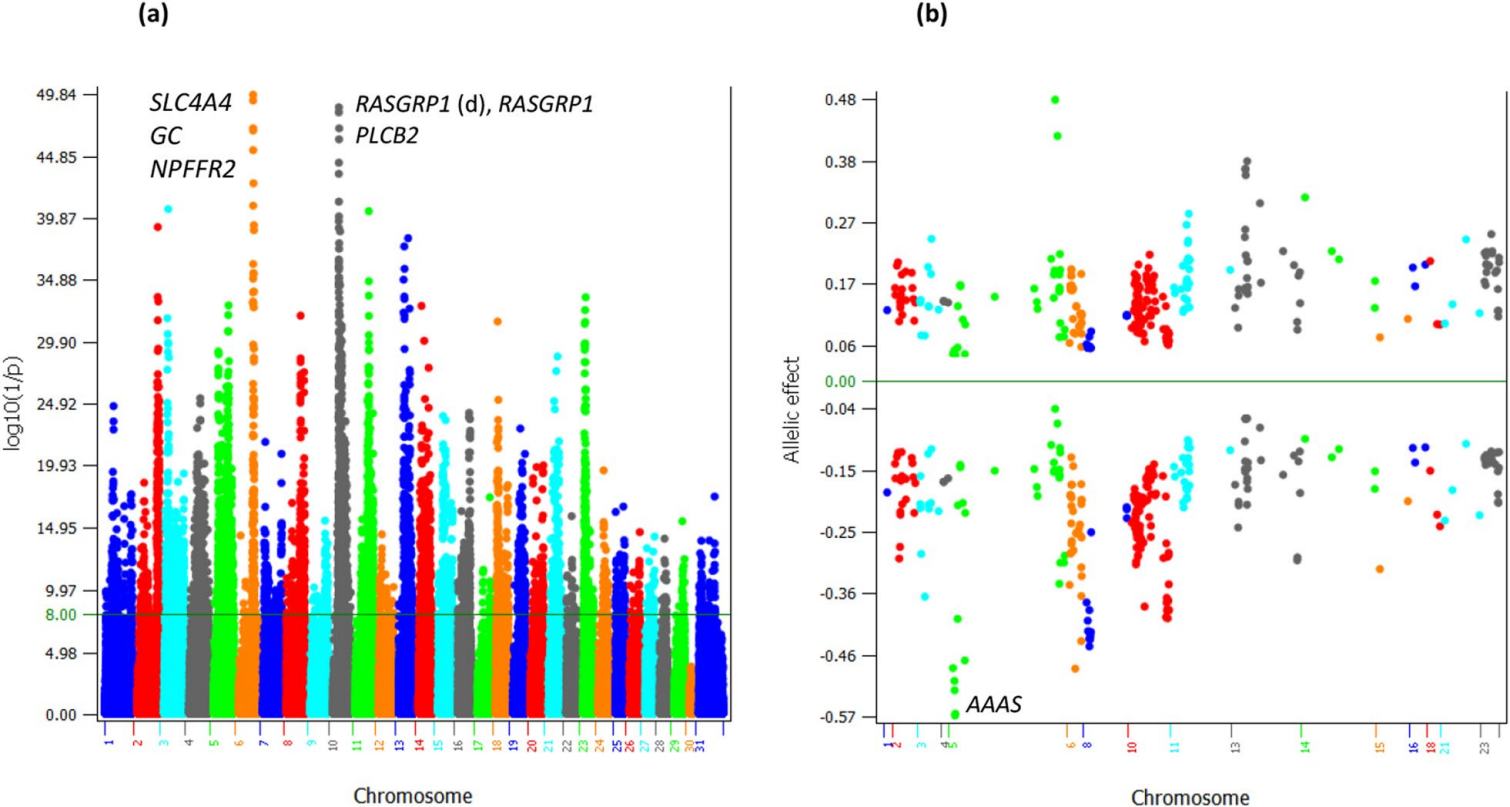
Additive effects of PL. **a** Manhattan plot of statistical significance of genome-wide additive effects. Chr30 is the pseudoautosome region and Chr31 is the X–Y nonrecombining region of the X chromosome. **b** Allelic effects of the top 300 additive effects

The GWAS detected 5390 significant additive effects distributed over all 29 autosomes and the X–Y nonrecombining region of the X chromosome (Chr31, Fig. 1a). Chr06 and Chr10 had the most significant effects followed by Chr11 and Chr13. Some of these effects were in or near genes known to affect production, reproduction, health, and immunity. The top 300 additive effects were distributed on sixteen chromosomes, including chromosomes 1, 2, 3, 4, 5, 6, 8, 10, 11, 13, 14, 15, 16, 18, 21 and 23 (see Additional file 1: Table S1). Among these 300 additive effects, negative allelic effects on average had larger effect sizes (absolute values), average − 0.218 for the negative alleles and 0.147 for the positive alleles. The *AAAS* gene of Chr05 had the most negative allelic effect (Fig. 1b), due to a sharply negative homozygous recessive genotype of *AAAS* (to be discussed). The dominance tests detected 38 dominance effects with log_10_(1/p) > 8 (see Additional file 2: Table S2), including some rare but sharply negative recessive effects. In the main text of this article, gene names mostly use gene symbols and the full gene names are given in the supplementary material (see Additional file 3: Table S3). In the descriptions and discussions below, the focus for additive effects is on Chr06 and Chr10 that had the largest clusters and most significant additive effects, and the focus for dominance effects is on chromosome regions with sharply negative homozygous recessive genotypes.

### Additive effects of Chr06

**Fig. 2:**
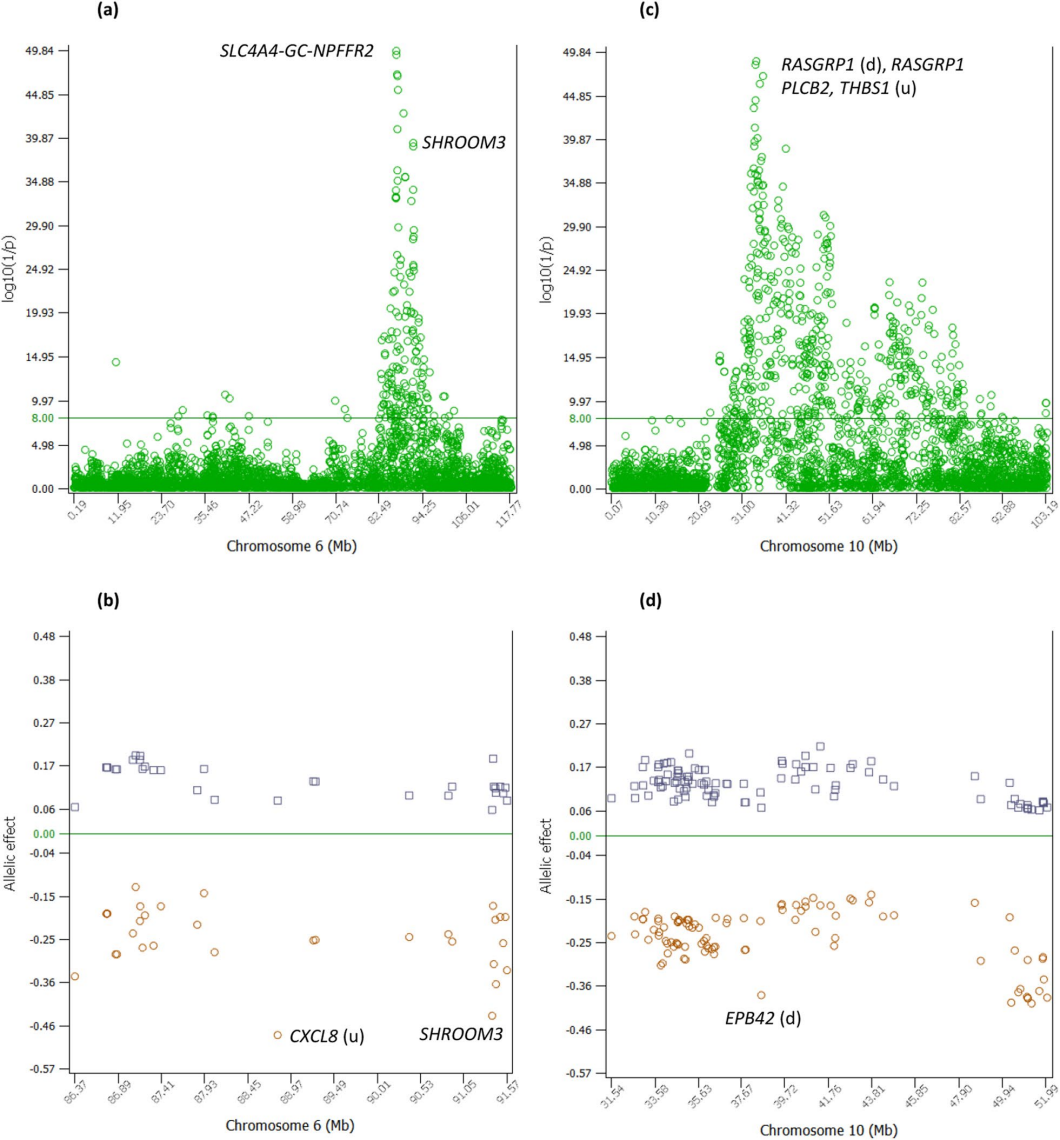
Additive effects of Chr06 and Chr10 for PL. **a** Statistical significance of additive effects of Chr06. **b** Allelic effects of Chr06 among the top 300 additive effects. **c** Statistical significance of additive effects of Chr10. **d** Allelic effects of Chr10 among the top 300 additive effects

The 86.39–91.57 Mb region of Chr06 had a cluster of additive effects (33 of the top 300 effects) with peak effects in the *SLC4A4-GC*-*NPFFR2* (SGN) region that was known to have highly significant effects for protein yield (PY), milk yield (MY), daughter pregnancy rate (DPR) and cow conception rate (CCR), and somatic cell score (SCS) [10, 21] (see Additional file 1: Table S1; Fig. 2a). This region had the most significant effects of all SNPs downstream of *SLC4A4* (#1 and #2 effects), the #5 and #7 effects between *GC* and *NPFFR2*, and the #9 effect in *NPFFR2.* The negative allelic effects had larger effect sizes (absolute values) than the positive allelic effects (Fig. 2b) and had allele frequencies of 0.356–0.598 (see Additional file 1: Table S1). The 90.90–91.57 Mb region had another cluster of ten significant additive effects among the top 300 additive effects, and the *SHROOM3* gene had nine of the ten effects including the #19 and #22 effects (see Additional file 1: Table S1; Fig. 2a). The likely reason for the highly significant PL effects in the SGN region is the previously reported highly significant effects for PY, DPR, CCR and SCS [21].

### Additive effects of Chr10

Chr10 had 95 of the top 300 effects distributed in a large region about 20 Mb in size (31.5–52 Mb) (Fig. 2c). The most significant Chr10 effects were in or near *RASGRP1* with the #3, #4 and #10 effects (Table 1). The *RASGRP1* gene activates the Erk/MAP kinase cascade and regulates T-cells and B-cells development, homeostasis and differentiation [24]. Given these important immunity functions, significant effects in or near *RASGRP1* for PL could be due to the immunity functions of *RASGRP1* contributing to the cow’s fitness and health, noting that *RASGRP1* was reported to be a candidate gene for mastitis resistance in Holstein cattle [25]. The negative allelic effects mostly had larger effect sizes (absolute values) than the positive allelic effects in the 32–36 Mb and 48–52 Mb regions, but the 38–44 Mb region mostly had symmetric effects where positive and negative effects had similar effect sizes (Fig. 2d). The frequencies of the negative alleles were in the range of 0.137–0.565 (see Additional file 1: Table S1). These results showed that the significant effects of the Chr10 region had more negative effects than positive effects and had substantial opportunities for increasing the frequencies of the positive alleles and reducing the frequencies of the negative alleles.

**Table 1:** Top 20 additive effects of productive life (PL)

SNP	Chr	Position	Candidate gene	Effect (α)	al +	ae+	f_al+	al–	ae–	f_al-	log_10_(1/p)
*rs133886272*	2	126,156,967	*LOC104971349-FAM46B*	− 0.42	2	0.137	0.673	1	− 0.282	0.327	39.18
*rs41255335*	3	23,565,194	*PHGDH*	0.39	1	0.184	0.529	2	− 0.207	0.471	40.63
*rs110527224*	6	86,860,291	*SLC4A4* (d)	− 0.45	2	0.158	0.649	1	− 0.293	0.351	49.84
*rs110380398*	6	86,877,334	*SLC4A4* (d)	0.45	1	0.158	0.649	2	− 0.293	0.351	49.38
*rs109452259*	6	87,068,809	*GC-NPFFR2*	0.42	1	0.181	0.572	2	− 0.242	0.428	47.15
*rs137147462*	6	87,153,414	*GC-NPFFR2*	− 0.39	2	0.181	0.539	1	− 0.212	0.461	40.91
*rs110434046*	6	87,184,768	*GC-NPFFR2*	− 0.44	2	0.159	0.635	1	− 0.277	0.365	46.96
*rs109034709*	6	87,316,810	*NPFFR2*	− 0.43	2	0.156	0.635	1	− 0.272	0.365	45.38
*rs109793149*	6	88,808,252	*CXCL8* (u)	0.57	1	0.082	0.857	2	− 0.490	0.143	42.71
*rs41588974*	6	91,406,353	*SHROOM3*	− 0.43	2	0.116	0.732	1	− 0.317	0.268	39.33
*rs137178400*	10	33,756,427	*TMCO5A-SPRED1*	− 0.41	2	0.173	0.581	1	− 0.240	0.419	43.48
*rs41647633*	10	33,940,919	*SPRED1*	− 0.43	2	0.121	0.717	1	− 0.307	0.283	39.66
*rs110578748*	10	34,000,269	*SPRED1* (d)	− 0.40	2	0.176	0.556	1	− 0.220	0.444	41.23
*rs110413607*	10	34,156,200	*RASGRP1*	− 0.44	2	0.177	0.595	1	− 0.259	0.405	48.43
*rs109718130*	10	34,176,744	*RASGRP1*	− 0.43	2	0.149	0.655	1	− 0.283	0.345	44.37
*rs110493658*	10	34,336,811	*RASGRP1* (d)	0.43	1	0.179	0.588	2	− 0.256	0.412	48.84
*rs136476033*	10	34,624,568	*LOC104973119-LOC104973122*	0.40	1	0.144	0.643	2	− 0.260	0.357	40.04
*rs134389993*	10	35,187,157	*THBS1* (u)	− 0.42	2	0.200	0.521	1	− 0.218	0.479	46.25
*rs137782429*	10	35,924,151	*PLCB2*	0.44	1	0.160	0.636	2	− 0.279	0.364	47.14
*rs110524929*	11	78,663,074	*SDC1-LAPTM4A*	0.41	1	0.268	0.343	2	− 0.140	0.657	40.47

‘d’ indicates the SNP is downstream of the gene. ‘u’ indicates the SNP is upstream of the gene. ‘α’ is the additive effect of the SNP as the difference between allelic effects of ‘allele 1’ and ‘allele 2’ (Eq. (10)) in months. ‘al + ’ is the positive allele, ‘al– ‘ is the negative allele, ‘ae + ’ is the allelic effect of the positive allele (Eq. (9)) in months, ‘ae**–**’ is the allelic effect of the negative allele (Eq. (9)) in months. ‘f_al + ’ is the frequency of the positive allele. ‘f_al-’ is the frequency of the negative allele

The observed additive effects indicated that multiple causal effects existed and that linkage disequilibrium (LD) between loci could not be the only reason for the multiple significant effects in the large Chr10 region. In several cases multiple insignificant effects were observed near highly significant effects (Fig. 2b). The *RASGRP1* gene had the #4 and #10 effects, but also had an insignificant effect (#8391) at 34,214,726, or 37,982 bp downstream of the #10 effect and 58,526 bp downstream of the #4 effect. Given the existence of insignificant effects near highly significant effects even within the same gene, LD with the significant effects in *RASGRP1* unlikely was the only reason for other significant additive effects further downstream of the 34,214,726 bp location with an insignificant effect. This is because the LD generally became weaker as the distance between the significant SNP in *RASGRP1* and a downstream SNP increased (see Additional file 4: Figure S1). The SGN region of Chr06 (Fig. 2a) had another striking example showing the limited LD effect on the statistical significance of an additive effect: an insignificant effect (#43,869) at 86,871,632 bp was between the #1 and #2 effects, 11,341 bp downstream of the #1 effect and 5702 bp upstream of the #2 effect. These results indicated that the current sample size could separate two SNPs within 5702–11,341 bp distances for their statistical significance on PL. The mixture of highly significant and insignificant effects in small regions provided evidence pointing to the limited role of LD in the number of significant effects and the likely existence of multiple causal effects on PL in the large Chr10 region. Prior to our study on PL, large chromosome regions with significant additive effects for milk production traits had been reported for Chr14, Chr06, Chr20, and Chr05 in Holstein cows [21].

### Additive effects of Chr14, Chr05 and Chr20 for PL

Milk production was another contributor to PL after fertility and health [2] and the previously reported highly significant PY effects of the SGN region of Chr06 could be a contributing factor to the highly significant PL effects of the SGN region. However, Chr06 was only one of the four chromosomes with highly significant SNP effects for milk production traits along with Chr14, Chr05, and Chr20 [21]. Given the significance of the SGN region for PL, the contributions of Chr14, Chr05 and Chr20 should be investigated.

**Fig. 3:**
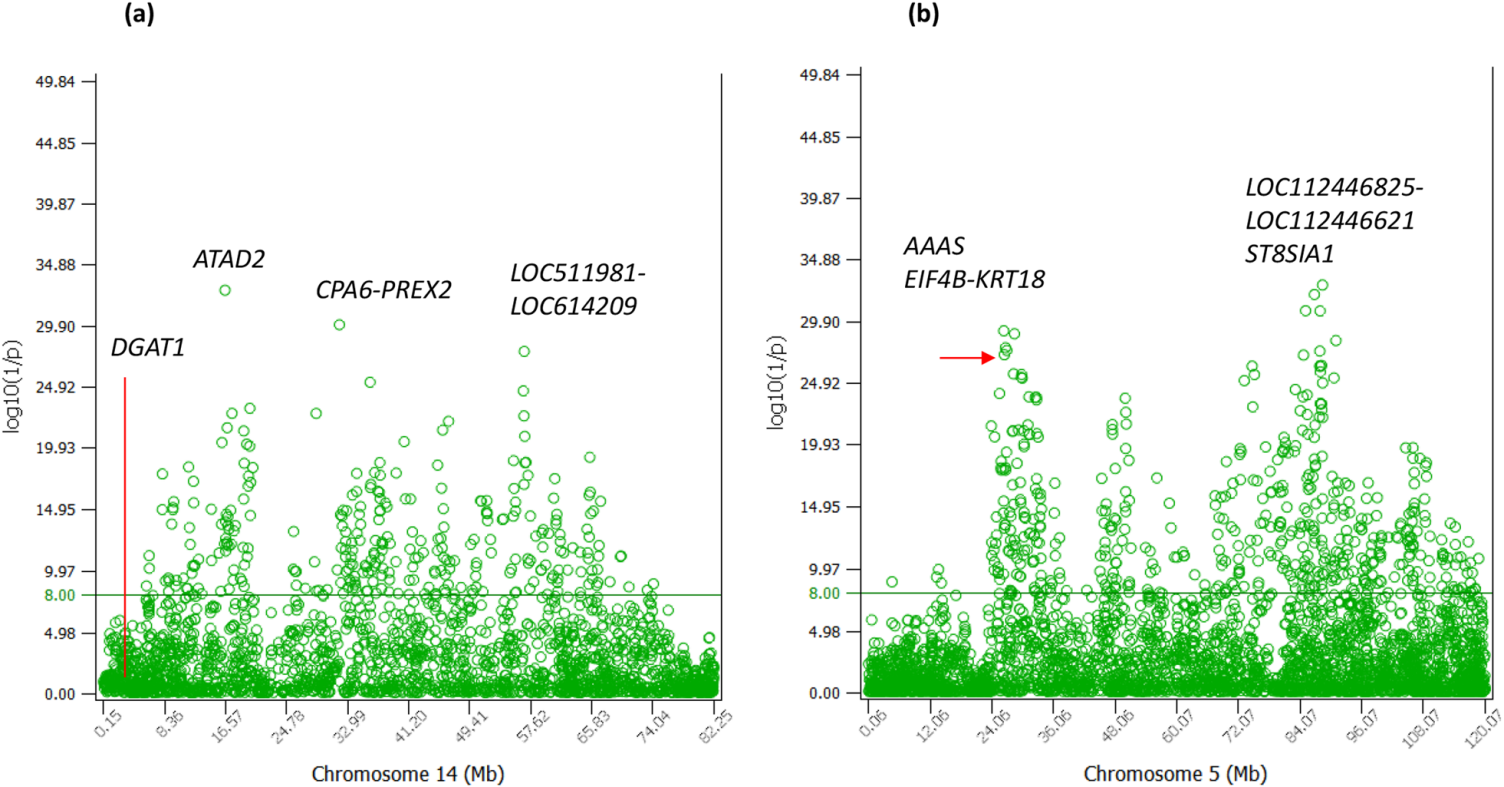
Additive effects of Chr14 and Chr05 for PL. **a** Statistical significance of additive effects of Chr14. **b** Statistical significance of additive effects of Chr05

The 0.46–0.89 Mb region of Chr14 containing *DGAT1* and about 0.43 Mb in size (based on ARS-UCD1.3 cattle genome assembly [17]) had the most significant additive effects for all five milk production traits, milk, fat and protein yields, and fat and protein percentages, considerably more significant than any other chromosome regions for these production traits [21], and interacted with all chromosomes for fat percentage [12, 26]. However, the entire 0.46–0.89 Mb region of Chr14 had no significant effects for PL, and the nearest significant effects ranking #2313 and #3029 were in the 6.28–6.32 Mb region, far from the 0.46–0.89 Mb region containing *DGAT1*. The *ATAD2* gene at 16.49 Mb and a large section of 32.99–68.83 Mb not known to have highly significant effects for the production traits had significant additive effects for PL with the best ranking #152 (Fig. 3a, see Additional file 1: Table S1). The lack of PL effects in the 0.46–0.89 Mb region containing *DGAT1* likely was due to the antagonism between fat yield (FY) and PY in that region. FY and PY currently have the largest relative emphasis in the net merit selection index, 21.8% for FY and 17% for PY [3, 4], and should be the most important contributing components to PL. SNP *rs109421300 (ARS-BFGL-NGS-4939*) in *DGAT1* had the most significant effects for all five production traits, MY, FY, PY, fat percentage (FPC) and protein percentage (PPC); and one allele of *rs109421300* had an extreme antagonism between FY and PY with the most positive allelic effect for FY and the most negative allelic effects for PY and MY [21]. Consequently, the positive contribution of FY and the negative contribution of PY to PL likely cancelled each other out, resulting in no significant effect for PL from this SNP. The antagonism between FY and PY extended to the entire 0.46–0.89 Mb region containing *DGAT1* in various degrees and this antagonism should be the reason for the lack of PL effects in this entire region.

Chr05 had highly significant effects for FY in *MGST1-SLC15A5*, *PLEKHA5*, *ABCC9* and *ST8SIA1* [21] and these gene regions had the #992, #217, #159 and #86 effects respectively, indicating that the highly significant effects of these genes for PL likely were due to their effects for FY, which currently has the largest relative emphasis in the net merit selection index [3, 4]. In addition, the 26.38–28.47 Mb region with *ATF7*, *AAAS*, *EIF4B* and *KRT18* genes had a cluster of significant additive effects for PL (see Additional file 1: Table S1; Fig. 3b). Interestingly, this region also had sharply negative recessive genotypes as to be described.

Chr20 had highly significant effects on MY in the *GHR-C6* region [21] but this region had only two significant effects for PL with the best ranking of #3363. Therefore, the highly significant effects of MY in the *GHR-C6* region of Chr20 did not result in significant effects for PL. Given that the MY effects of Chr20 were more significant than those of the SGN region of Chr06 [21], the MY effects of the SGN region unlikely contributed to the highly significant PL effects in the SGN region.

The PL results of the four chromosome regions with most significant effects for milk production traits showed that only the SGN region of Chr06 with highly significant effects for PY and the Chr05 regions with highly significant effects for FY had highly significant effects for PL. In contrast, the Chr20 region with highly significant effects for MY only had significant effects for PL that were not ranked high and the Chr14 region with highly significant effects for all five production traits had no significant effects for PL.

### Dominance effects of PL

**Fig. 4:**
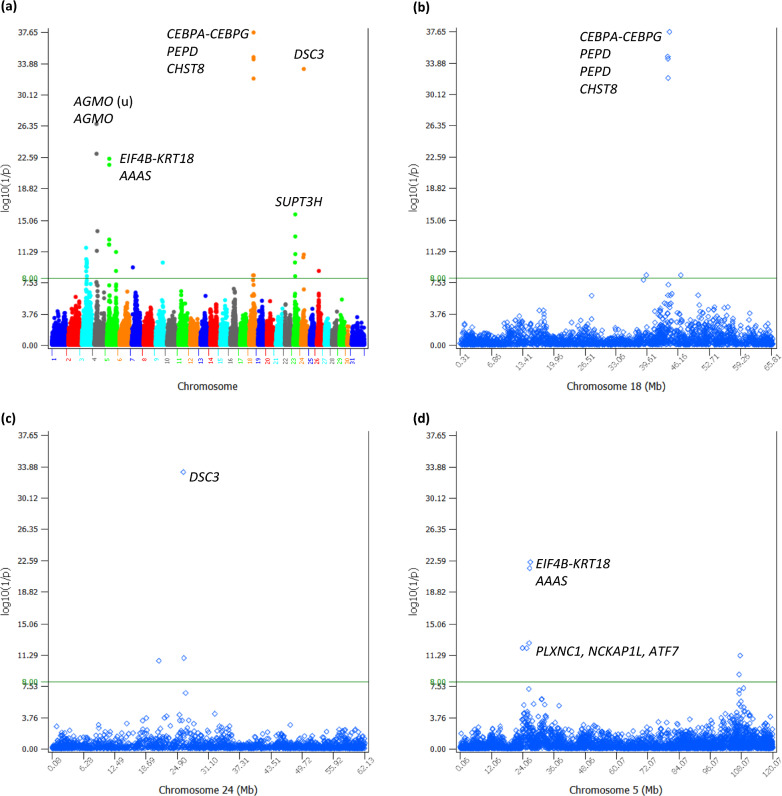
Dominance effects of PL. **a** Manhattan plot of statistical significance of genome-wide dominance effects. Chr30 is the pseudoautosome region of the X chromosome and Chr31 is the X–Y nonrecombining region of the X chromosome. **b** Statistical significance of dominance effects of Chr18. **c** Statistical significance of dominance effects of Chr24. **d** Statistical significance of dominance effects of Chr05

**Fig. 5:**
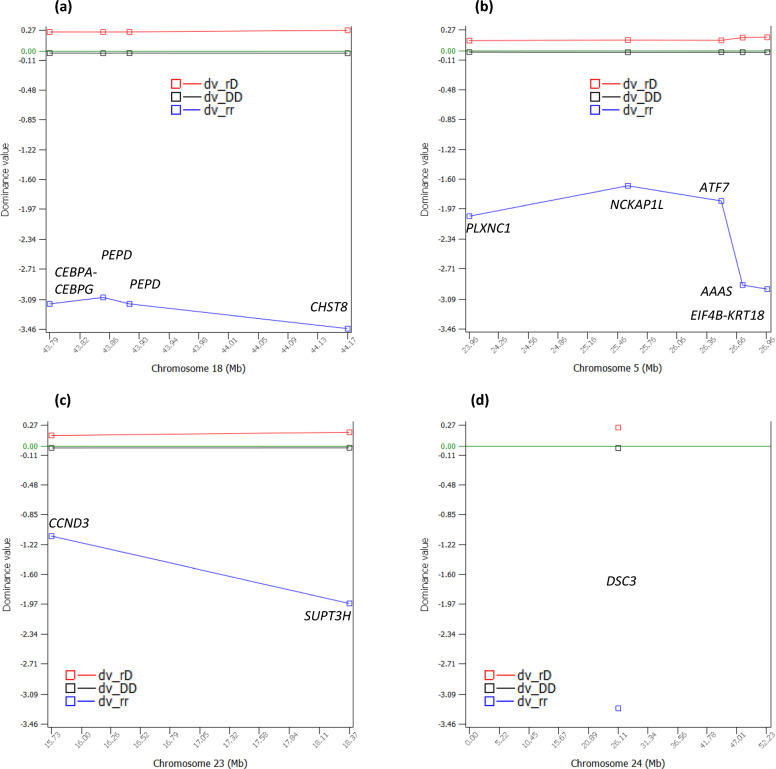
Dominance values of four chromosomes with sharply negative recessive genotypes. **a** Dominance values of four Chr18 SNPs. **b** Dominance values of five Chr05 SNPs. **c** Dominance values of two Chr23 SNPs. **d** Dominance values of a Chr24 SNP. ‘dv_DD’ is the dominance value of the homozygous genotype with two dominant alleles (DD). ‘f_DD’ is the frequency of the homozygous genotype of the dominant allele. ‘dv_rD’ is the dominance value of the heterozygous genotype with one dominant allele (D) and one recessive allele (r). ‘f_rD’ is the frequency of the heterozygous genotype. ‘dv_rr’ is the dominance value of the homozygous genotype with two recessive alleles (rr). ‘f_rr’ is the frequency of the homozygous genotype of the recessive allele

**Fig. 6:**
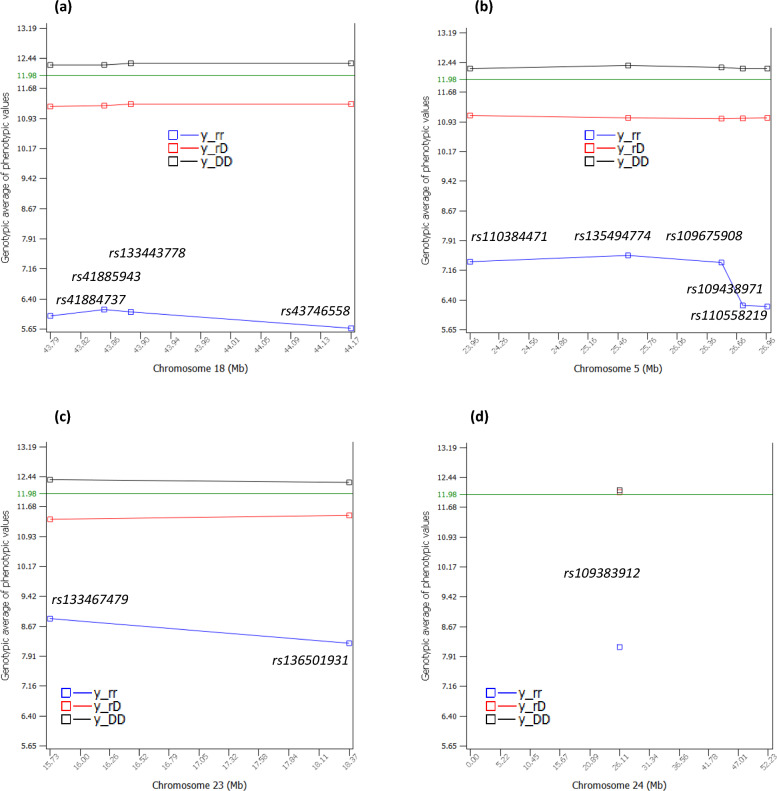
Genotypic avarages of phenotypic values of four chromosomes with sharply negative recessive genotypes. **a** Genotypic averages of the phenotypic values of four Chr18 SNPs. **b** Genotypic averages of the phenotypic values of five Chr05 SNPs. **c** Genotypic averages of the phenotypic values of two Chr23 SNPs. **d** Genotypic averages of the phenotypic values of a Chr24 SNP. y_ij = genotypic average of the phenotypic values of cows with the ij SNP genotype, where i or j = r indicates the recessive allele, and i or j = D indicates the dominant allele. The green horizontal line of ‘11.98’ is the average of the phenotypic values of all cows

**Fig. 7:**
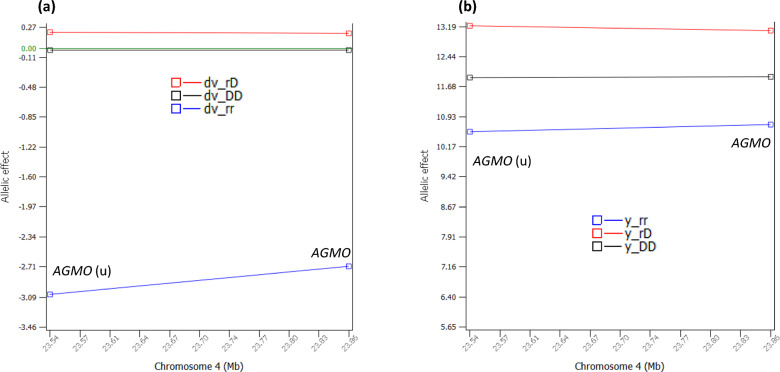
Two Chr04 SNPs with heterozygous advantage for both dominance and phenotypic values. **a** Dominance values of two Chr04 SNPs. **b** Genotypic averages of the phenotypic values of two Chr04 SNPs. dv_DD’ is the dominance value of the homozygous genotype with two dominant alleles (DD). ‘f_DD’ is the frequency of the homozygous genotype of the dominant allele. ‘dv_rD’ is the dominance value of the heterozygous genotype with one dominant allele (D) and one recessive allele (r). ‘dv_rr’ is the dominance value of the homozygous genotype with two recessive alleles (rr). ‘f_rr’ is the frequency of the homozygous genotype of the recessive allele. y_ij = genotypic average of the phenotypic values of cows with the ij SNP genotype, where i or j = r indicates the recessive allele, and i or j = D indicates the dominant allele

The dominance tests detected 38 significant dominance effects of PL with log_10_(1/p) > 8 (see Additional file 2, Table S2; Fig. 4a). Four SNPs of Chr18 in a 381.389 Kb region had the most significant dominance effects (Fig. 4b), followed by a SNP of Chr24 (Fig. 4c), and two SNPs of Chr05 in a 248.719 Kb region (Fig. 4d). Among the 38 significant dominance effects, 12 dominance effects involved sharply negative homozygous recessive genotypes for PL (Table 2), including the dominance effects of four SNPs in *CEBPA-CEBPG*, *PEPD* and *CHST8* of Chr18, five SNPs in *EIF4B-KRT18*, *AAAS*, *PLXNC1*, *NCKAP1L* and ATF7 of Chr05, two SNPs in *CCND3* and *SUPT3H* of Chr23, and one SNP in *DSC3* of Chr24 (Table 2, Fig. 5a–d). For these SNPs, only the heterozygous genotypes had positive dominance values, the homozygous dominance genotypes had slightly negative dominance values, and the homozygous recessive genotypes had sharply negative dominance values, where the dominance values were defined by Eq. (12) that removed additive values from the phenotypic values. The recessive genotypes all had low frequencies of 0.005–0.012, affecting 4637–13,285 cows. An allele was defined as a dominant allele if this allele neutralized the effect of the alternative allele completely or partially when in heterozygous status. Similarly, an allele was defined as a recessive allele if the effect of this allele was neutralized by the alternative allele completely or partially when in heterozygous status. Based on the dominance values, all the 38 dominance effects (see Additional file 2: Table S2) were slight overdominance effects because the dominance values of the heterozygous genotypes were more extreme than the dominance values of the two homozygous genotypes. Based on the phenotypic values that contain both additive and dominance values, most SNPs with significant dominance effects had partial dominance where the average of the heterozygous genotype was below that of the homozygous dominant genotype but was much higher than that of the homozygous recessive genotype (Fig. 6). The two Chr04 SNPs were the only example of overdominance in terms of both dominance values and genotypic averages of the phenotypic values and were the only examples of heterozygous advantage at the phenotypic level (Fig. 7).

**Table 2:** Dominance effects of SNPs with sharply negative recessive genotypic averages for PL

SNP	Chr	Position	Candidate gene	D	r	y_DD	f_DD	y_rD	f_rD	y_rr	f_rr	Effect (δ)	log_10_(1/p)	NI
*rs111023007*	4	23,542,497	*AGMO* (u)	*C*	*A*	11.89	0.858	13.19	0.137	10.53	0.005	1.75	26.62	− 2.00
*rs29023731*	4	23,863,959	*AGMO*	*A*	*C*	11.91	0.852	13.07	0.143	10.71	0.005	1.56	23.01	− 1.77
*rs110384471*	5	23,955,328	*PLXNC1*	*G*	*A*	12.26	0.858	11.07	0.138	7.36	0.005	1.17	12.08	− 4.31
*rs135494774*	5	25,556,149	*NCKAP1L*	*G*	*A*	12.34	0.831	11.01	0.162	7.52	0.007	0.99	12.06	− 4.15
*rs109675908*	5	26,499,453	*ATF7*	*A*	*G*	12.29	0.841	10.99	0.153	7.34	0.006	1.07	12.67	− 4.30
*rs110558219*	5	26,715,326	*AAAS*	*G*	*A*	12.26	0.859	11.00	0.137	6.25	0.004	1.64	21.69	− 5.38
*rs109438971*	5	26,964,045	*EIF4B-KRT18*	*A*	*G*	12.26	0.859	11.01	0.137	6.22	0.004	1.66	22.42	− 5.41
*rs41884737*	18	43,786,051	*CEBPA-CEBPG*	*G*	*A*	12.25	0.851	11.21	0.143	5.96	0.006	1.83	34.67	− 5.77
*rs41885943*	18	43,854,199	*PEPD*	*G*	*A*	12.25	0.848	11.23	0.146	6.12	0.006	1.79	34.41	− 5.62
*rs133443778*	18	43,887,966	*PEPD*	*G*	*A*	12.29	0.851	11.27	0.143	6.06	0.006	1.83	32.09	− 5.72
*rs43746558*	18	44,167,440	*CHST8*	*G*	*A*	12.29	0.855	11.27	0.14	5.65	0.005	2.01	37.65	− 6.13
*rs133467479*	23	15,731,441	*CCND3*	*G*	*A*	12.34	0.792	11.34	0.196	8.84	0.012	0.71	9.90	− 3.00
*rs136501931*	23	18,370,790	*SUPT3H*	*G*	*A*	12.27	0.84	11.44	0.153	8.22	0.007	1.17	15.70	− 3.63
*rs109383912*	24	26,114,907	*DSC3*	*G*	*A*	12.09	0.858	12.04	0.137	8.13	0.005	1.88	33.25	− 3.94

‘u’ indicates the SNP is upstream of the gene. ‘r’ is the recessive allele. ‘D’ is the dominant allele. ‘y_DD’ is the average of phenotypic values of cows with the homozygous genotype of the dominant allele (DD) in months. ‘f_DD’ is the frequency of the homozygous genotype of the dominant allele. ‘y_rD’ is the average of phenotypic values of cows with the heterozygous genotype (rD) in months. ‘f_rD’ is the frequency of the heterozygous genotype. ‘y_rr’ is the the average of phenotypic values of cows with the homozygous genotype of the recessive alleles (rr) in months. ‘f_rr’ is the frequency of the homozygous genotype of the recessive allele. ‘δ’ is the dominance effect of the SNP as the difference between the heterozygous dominance value and the average of the two homozygous dominance values (Eq. (11)). ‘NI’ is the negative impact of Eq. (13) in months

### Heifer culling for recessive genotypes

These 12 SNPs of Chr18, Chr05, Chr24 and Chr23 with sharply negative recessive genotypes for PL (Table 2) should be considered for eliminating heifers carrying the homozygous recessive genotypes to avoid heifers with poor PL performance. To evaluate the phenotypic impact of heifer elimination using these SNPs, we compared the genotypic averages of the phenotypic values for these SNPs.

The negative impact (NI) of a recessive genotype was calculated as the difference between mean phenotypic values of cows with the homozygous recessive genotype and the mean values of the other two genotypes, the heterozygous and homozygous dominant genotypes (Eq. (13)). The NI values of the 12 SNPs for PL were between − 3.0 and − 6.13 months (Table 2). The most negative recessive genotypes were those of the four Chr18 SNPs with NI values between − 5.41 and − 6.13 months (Fig. 6a, Table 2), followed by five Chr05 SNPs with NI values between − 4.15 and − 5.77 months (Fig. 6b, Table 2), two SNPs of Chr23 with NI values of − 3.0 and − 3.63 months, (Fig. 6c, Table 2), and one SNP of Chr24 with NI value of − 3.94 months (Fig. 6d, Table 2). Of these sharply negative recessive genotypes for PL, the four Chr18 SNPs (Fig. 6a) had sharply negative recessive effects for DPR and CCR, two of the Chr05 SNPs (*rs109438971* and *rs110558219*) (Fig. 6b) had sharply negative recessive effects for age at first calving (AFC), and all these SNPs were also negative for milk, fat and protein yields and were recommended for the elimination of heifers carrying the recessive genotype for any of these SNPs [10, 11]. The PL results in this study added another reason for eliminating the recessive genotypes of those six SNPs in heifers.

The two Chr04 SNPs with highly significant dominance effects could also be considered for eliminating heifers with the recessive genotype for any of the two SNPs, but the NI values of these two SNPs for PL (− 1.77 to − 2.0 months, Fig. 7, Table 2) were not as negative as those of the other 12 SNPs. The recessive genotypes of these two SNPs were also sharply negative for heifer conception rate (HCR) but were slightly positive for milk, fat and protein yields [10]. On balance between the sharply negative recessive effects for HCR, substantially negative recessive effects for PL, and slightly positive effects for the yield traits, the recessive genotypes of the two Chr04 SNPs should be used for heifer culling. However, we are not including the two Chr04 SNPs in our recommendation for heifer culling because whether avoiding the sharply negative effects for HCR or having slightly positive yield traits is more important should be decided by the farmers or breeders. If the two Chr04 SNPs are also used for heifer culling, the total number of SNPs for heifer culling increases from 12 to 14.

### Bull culling and SNP-guided mating

The information about the sharply negative recessive genotypes in this study can be used for mating plans to avoid producing recessive heifers using the methods of bull culling and SNP-guided mating. These two methods to avoid recessive genotypes in the next generation involve more complicated issues than heifer culling for the recessive genotypes for any of the 12 SNPs we recommend.

Bull culling essentially eliminates bulls carrying the recessive allele for any of the 12 SNPs with sharply negative recessive genotypes but is more complicated than heifer culling in terms of consequences, and the value of bull culling is questionable. A bull with a recessive genotype passes a recessive allele to the daughters with 100% probability but only a small fraction of the daughters is expected to have the damaging recessive genotype because the frequency of the recessive allele is small, 0.073–0.097 (see Additional file 2: Table S2), meaning that only 7–10% of the daughters of a homozygous recessive sire are expected to have the damaging recessive genotype. For a bull with a heterozygous genotype carrying the recessive allele, the bull passes the recessive allele to the daughters with a 50% probability, resulting in only 3–5% of the daughters with the damaging recessive genotype. If the daughters are genotyped with the SNPs, the recessive daughters can be culled as soon as the SNP genotypes become available. Given that the majority of the daughters of a sire carrying the recessive allele are unaffected by the recessive allele at the phenotypic level, the value of bull culling is questionable. Yet, other factors also need to be considered, including the total genetic merit of the bull, and the number of bulls that can be culled without resulting in a shortage of breeding bulls.

SNP-guided mating uses the SNP genotypes of the sires and dams to select breeding pairs that do not produce the homozygous recessive offspring. Under the assumption of culling heifers with the recessive genotypes, a heifer with the recessive genotype for any of the SNPs for heifer culling does not have a chance to be part of the breeding population. Therefore, only heifers with the dominant and heterozygous genotypes (DD and rD) are potential dams of the next generation, and a bull with the homozygous recessive genotype (rr) or the heterozygous genotype (rD) may not be mated with a rD dam. The breeding pairs (sire × dam) that do not produce the homozygous recessive daughters are DD × DD, DD × rD, rD × DD, and rr × DD. This type of SNP-guided mating is less severe than bull culling because sires carrying the recessive alleles are allowed to produce the next generation without producing homozygous recessive daughters. However, the feasibility of SNP-guided mating for all 12 SNPs requires real-data evaluation.

### Fine mapping

Results of this study showed the power of over one million cows for fine mapping and the limitation of the current SNP density of fewer than 80,000 SNPs. For all the 38 SNPs with significant dominance effects, both their flanking SNPs were insignificant and the average distance between a significant SNP and an insignificant flanking SNP was 23,545 bp (see Additional file 5: Table S4). The closest insignificant flanking SNP (*rs136246450* with #1047 effect) next to a significant SNP (*rs110558219* with #9 effect) was only 2774 bp apart. This example showed the power of fine mapping of the sample size of 1,103,641 Holstein cows, an ability to distinguish the significance levels between two SNPs as close as 2774 bp apart. The largest distance between an insignificant flanking SNP and a significant SNP was 113,506 bp, which was the distance between *rs134764130* with the #50 effect (insignificant) and *rs109675908* with the #13 effect, showing the limitation of the SNP density of fewer than 80,000 SNPs in terms of SNP spacing. Assuming an average of 23,545 bp spacing between a significant non-causal SNP and the underlying causal variant, the non-causal SNP and the causal variant should have strong correlations. For example, the correlation between *rs109718130* and two SNPs that were 20,544 bp and 180,611 bp downstream was 0.89 and 0.92 respectively. Therefore, elimination of the SNP recessive genotypes detected in this study should be able to eliminate most of the true causal genotypes even if none of the significant SNPs was a causal SNP.

## Conclusions

The million-cow GWAS for PL identified two chromosome regions with the most significant additive effects for PL: the SGN region of Chr06 that was known to have highly significant effects for two fertility traits, milk yield and somatic cell score; and a large Chr10 region with multiple genes with important immunity functions. Rare but sharply negative homozygous recessive genotypes for PL existed. Four Chr18 SNPs, five Chr05 SNPs, two Chr23 SNPs and a Chr24 SNP were sharply negative for PL, and these twelve SNPS were recommended for eliminating heifers with the homozygous recessive genotype for any of the twelve SNP. The results of this study provided high-confidence evidence for the understanding of genetic factors affecting PL.

## Supplementary Information

**Additional file 1: Table S1.** Top 300 SNP additive effects of PL. This table provides test results for the top 300 additive effects. ‘d’ indicates the SNP is downstream of the gene. ‘u’ indicates the SNP is upstream of the gene. ‘Allele-1’ is the nucleotide of allele ‘1’, and ‘Allele-2’ is the nucleotide of allele ‘2’. ‘al+’ is the positive allele, ‘al–’ is the negative allele, ‘ae+’ is the allelic effect of the positive allele (Eq. (9)), ‘ae**–**’ is the allelic effect of the negative allele (Eq. (9)). ‘f_al+’ is the frequency of the positive allele. ‘f_al-’ is the frequency of the negative allele. ‘Effect’ is the additive effect of the SNP as the difference between allelic effects of allele ‘1’ and allele ‘2’ (Eq. (10)).

**Additional file 2: Table S2.** SNPs with significant dominance effects of PL. This table provides test results for all significant dominance effects. ‘Allele-1’ is the nucleotide of allele ‘1’, and ‘Allele-2’ is the nucleotide of allele ‘2’. ‘Effect’ is the dominance effect of the SNP as the difference between the heterozygous dominance value and the average of the two homozygous dominance values (Eq. (11)). ‘Yij’ is the genotypic average of phenotypic values of cows with the ij genotype (i,j = 1,2). ‘dl++’ is the genotype with the highest dominance value. ‘dv++’ is the dominance value of the ‘dl++’ genotype. ‘dl+–’ is the genotype with the second highest dominance value. ‘dv+–’ is the dominance value of the ‘dl+–’ genotype. ‘dl–’ is the genotype with the lowest dominance value. ‘dv–’ is the dominance value of the ‘dl–’ genotype. ‘f_dl++’ is the frequency of the dl++ genotype. ‘f_dl+–’ is the frequency of the ‘dl + –’ genotype. ‘f_ dl–’ is the frequency of the ‘dl–' genotype. ‘ae+’ is the allelic effect of the positive allele (Eq. (9)). ‘ae**–**’ is the allelic effect of the negative allele (Eq. (9)). ‘f_al+’ is the frequency of the positive allele. ‘f_al-’ is the frequency of the negative allele. The 12 SNPs on the sheet ‘12SNPs’ are recommended for heifer culling.

**Additional file 3: Table S3.** Gene names of candidate genes with additive and dominance effect described in the main text. This table provides full gene names of gene symbols discussed in the main text.

**Additional file 4: Figure S1.** Linkage disequilibrium (LD) between *rs110413607* of *RASGRP1* and the remaining 94 SNPs with the top-300 additive effects. This figure shows the LD became weaker as the distance between the SNP and *rs110413607* became larger.

**Additional file 5: Table S4.** SNPs with sharply negative recessive genotypes and flanking SNPs for PL. This table provides test results for all significant dominance effects. Each significant dominance effect has two flanking SNPs with insignificant dominance effects showing the power of fine mapping of the large sample size of this study. ‘Allele-1’ is the nucleotide of allele ‘1’, and ‘Allele-2’ is the nucleotide of allele ‘2’. ‘Effect’ is the dominance effect of the SNP as the difference between the heterozygous dominance value and the average of the two homozygous dominance values (Eq. (11)).

## Data Availability

The original genotype data are owned by third parties and maintained by the Council on Dairy Cattle Breeding (CDCB). A request to CDCB is necessary for getting data access on research, which may be sent to: João Dürr, CDCB Chief Executive Officer (joao.durr@cdcb.us). All other relevant data are available in the manuscript and Additional Files.
