# Supplementary figures and images for: A million-cow genome-wide association study of productive life in U.S. Holstein cows
Source: Genet Sel Evol. 2024 Sep 26;56:67. doi: 10.1186/s12711-024-00935-1 (PMC11426094; doi:10.1186/s12711-024-00935-1)

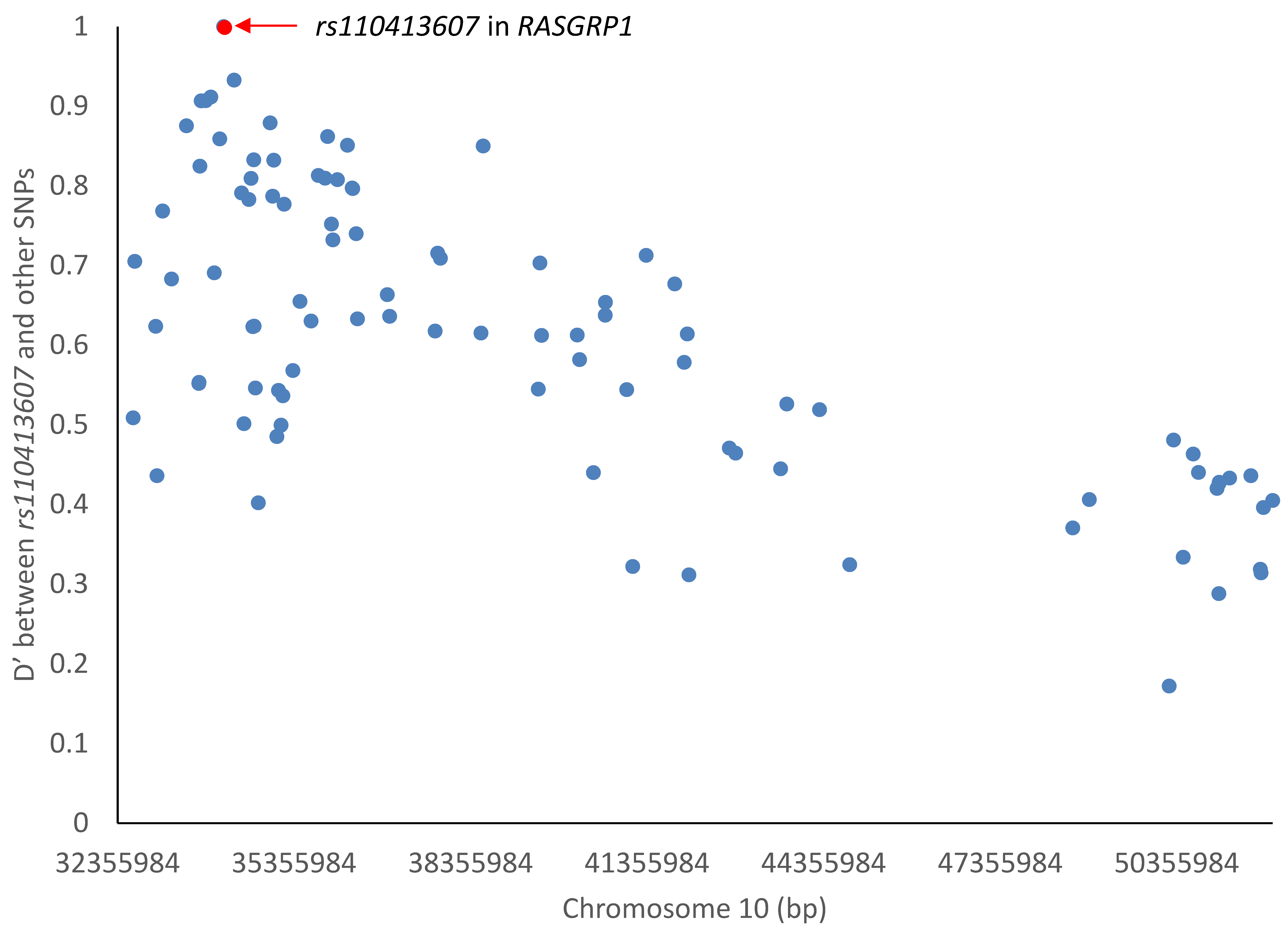

Supplement: Supplementary file 4 — Additional file 4: Figure S1. Linkage disequilibrium (LD) between rs110413607 of RASGRP1 and the remaining 94 SNPs with the top-300 additive effects. This figure shows the LD became weaker as the distance between the SNP and rs110413607 became larger. [file 12711_2024_935_MOESM4_ESM.tif]
